# Developing SENSES: Student experience of non-shared environment scales

**DOI:** 10.1371/journal.pone.0202543

**Published:** 2018-09-06

**Authors:** Sundus Yerdelen, Tracy Durksen, Kaili Rimfeld, Robert Plomin, Kathryn Asbury

**Affiliations:** 1 Faculty of Education, Kafkas University, Kars, Turkey; 2 School of Education, Faculty of Arts and Social Sciences, UNSW Australia, Sydney, NSW, Australia; 3 Social, Genetic and Developmental Psychiatry Centre, Institute of Psychiatry, King’s College London, London, United Kingdom; 4 Psychology in Education Research Centre, Department of Education, University of York, York, United Kingdom; Leibniz Institute for Educational Trajectories, GERMANY

## Abstract

Twin and adoption studies find that non-shared environmental (NSE) factors account for variance in most behavioural traits and offer an explanation for why genetically identical individuals differ. Using data from a qualitative hypothesis-generating study we designed a quantitative measure of pupils’ non-shared experiences at the end of formal compulsory education (SENSES: Student Experiences of Non-Shared Environment Scales). In *Study 1* SENSES was administered to *n* = 117 16–19 year old twin pairs. Exploratory Factor Analysis yielded a 49-item 10 factor solution which explained 63% of the variance in responses. SENSES showed good internal consistency and convergent and divergent validity. In *Study 2* this factor structure was confirmed with data from *n* = 926 twin pairs and external validity was demonstrated via significant correlations between 9 SENSES factors and both public examination performance and life satisfaction. These studies lend preliminary support to SENSES but further research is required to confirm its psychometric properties; to assess whether individual differences in SENSES are explained by NSE effects; and to explore whether SENSES explains variance in achievement and wellbeing.

## Introduction

It has long been established by twin and adoption studies that non-shared environmental (NSE) effects explain variance in most behavioural and psychological traits, particularly after the preschool years e.g. [1, 2, 3]. NSE effects are those that make siblings brought up together differ from each other. They are uncorrelated with genetic effects and, for this reason, represent potentially interesting targets for intervention. However, it has proved almost as difficult to identify the specific experiences that can explain NSE variance as it has been to identify the specific genes that explain genetic variance [4]. We have both a ‘missing heritability’ and a ‘missing environments’ problem [5,6]. The current study was motivated by a need to identify measured environments that can explain variance attributable to NSE in educationally relevant behaviour, such as achievement and wellbeing, with a view to possible intervention.

It has been difficult to identify NSE experiences because NSE effects, like genetic effects, tend to be many, small and involved in dynamic relationships with genes and other experiences. A further difficulty is that NSE effects include measurement error and one possibility is that they represent nothing but measurement error. However, this seems unlikely as some specific NSE effects have been found to explain small proportions of variance and, indeed, to show a degree of stability over time [7]. The largest body of research in this area has focused on parenting and has found effects that explain small proportions of variance [8] and which, in some studies, explain more variance at the extremes [9]. However, it remains possible that the measures of non-shared environment used in prior research do not explain much NSE variance because they do not accurately measure individuals’ experiences. It is important, therefore, to take a closer look at students’ experiences of the world in which they learn, and their perceptions of those experiences.

To that end, the current study was preceded by a qualitative hypothesis-generating MZ twin differences study designed to explore in detail the non-shared experiences of young people preparing for the public examinations (General Certificates of Secondary Education: GCSEs) taken by most UK pupils at age 16 [10, 11]. The focus was on MZ twins because behavioural or psychological differences between MZ twins cannot be explained by shared genes (because they can be assumed to have identical genotypes, albeit with a small chance of mutation) or shared environmental effects, and must therefore be explained by NSE effects, including measurement error. By asking MZ twins about differences between them in educationally relevant behaviour we were able to develop testable hypotheses about potential NSE influences at a transitional time, the point at which UK pupils make choices about further education, training and employment. The educationally-relevant traits we focused on were achievement and wellbeing (life satisfaction) as these are particularly salient variables at a time when young people are making important, potentially life-changing, decisions about their next steps in education or employment. Emerging hypotheses related to factors including perceived differences in teacher quality, teacher-pupil relationships and individual effort. Although psychology has already identified such factors as important correlates of achievement, this study was novel in suggesting that they may explain NSE variance specifically. The current study was designed to develop a quantitative measure that would make it possible to test these genetically-informed hypotheses.

We know that NSE factors, including measurement error, explain one-fifth of the variance in GCSE performance [12,13]. We therefore expected that families’ explanations of why one twin performed better than the other in their examinations could explain a maximum of 20% of variation in exam performance, and probably considerably less given that measurement error is likely to play a significant role [14]. The study also focused on participants’ well-being and we know that there is more NSE variance to be explained here. In a recent meta-analysis, for instance, Bartels [15] found that genetic effects explained 32% of the variance in self-reported life satisfaction and 36% of the variance in feelings of well-being. The remaining variance in both types of measure was explained by non-shared environmental factors. Identifying NSE influences on well-being therefore represents an important challenge.

The current research had two main aims: (1) to design a measure that reflected qualitative accounts of NSE experience at this transitional time; and (2) to assess the factor structure, reliability and validity of this newly developed measure. We aimed to make a useful contribution to research in this area by developing a measure that can explain a proportion of environmental variance in educationally relevant traits in late adolescence. Because NSE effects are uncorrelated with genetic effects such a measure may feasibly form a useful basis for environmental intervention in the future.

## Study 1

### Aims

The aims of Study 1 were threefold:

■ To develop an item pool based on data collected in an earlier qualitative phase of the project.
■ To reduce the number of items needed to measure experiences that may explain NSE variance in educationally-relevant outcomes.
■ To extract underlying factors and assess reliability.

### Method

#### Participants

Participants were drawn from the UK Twins’ Early Development Study (TEDS). TEDS is an on-going longitudinal study of three cohorts of twins born in 1994, 1995 and 1996 (16). The TEDS sample has been shown to be reasonably representative of the UK population of same-age adolescents and their parents [16, 17]. 300 twin pairs were invited to take part and data were gathered from *n* = 115 pairs and 2 unpaired twins (*n* = 117) who provided informed consent, 58 dizygotic pairs (62% female) and 57 monozygotic pairs (58% female) plus one dizygotic male twin and one monozygotic female twin. Twins were provided with a detailed information sheet and questionnaire completion indicated their consent to participate. Data were subsequently received from a further 6 pairs, but too late to be incorporated into analyses. Participants’ ages ranged from 16 to 19 (M = 18.28).

#### Measure development and procedure

In an earlier phase of this project we gathered qualitative questionnaire data from *n* = 497 pairs of MZ twins (61% female) and interview data from *n* = 95 of these pairs (10–11). These twin pairs were all participants in TEDS and were all aged between 16 and 19 (M = 17.3). Both questionnaires and interviews asked MZ pairs, and one parent from each family, to describe and explain differences between them in a range of traits including GCSE achievement and wellbeing. This represented an attempt to generate new hypotheses about NSE influences on young people approaching the end of their formal compulsory education.

We drew on this rich dataset to build up an item bank for the current study. We prepared 175 draft items and revised them after conducting a small feasibility study with *n* = 6 young people aged 16–19. We then administered the items to our Study 1 sample of *n* = 117 twin pairs (*n* = 234 individuals). This initial questionnaire aimed to comprehensively represent the breadth and depth of our qualitative data and was therefore very long. We engaged in a process of extensive data reduction once the data were collected.

We organised our data into two related samples, Sample 1 and Sample 2, with one twin from each pair (randomly selected) represented in each. We identified items that could be excluded on the basis of data from both samples. More specifically, items were excluded for the following reasons:

■ Violations of univariate normality i.e. skewness or kurtosis values outside of the range -2 to +2.
■ Low correlations within the expected area (r < 0. 20).
■ Multi-collinearity.
■ Not adequately representing the qualitative data e.g. only mentioned by a small number of participants.

This initial data reduction process allowed us to discard 93 items, leaving 82. We then conducted Principal Component Analysis (PCA) with Sample 1 for the sole purpose of further data reduction, that is, not for factor extraction [18]. PCA suggested the exclusion of 33 further items on grounds of cross-loadings, that is, items having a loading of 0.4 or higher on more than one component [19] and also having a lower than 0.2 loading difference between the primary and alternative factors [20]; or the clustering of fewer than three items i.e. too few to constitute a viable factor [21]. This data reduction process left us with 49 items with which to measure NSE influences on young people preparing to leave school (the PCA process suggested using 48 items and the reasons for retaining 49 are discussed later). All items used a 5-point Likert response scale ranging from 1 = ‘not at all true’ to 5 = ‘very true’. These 49 items make up the SENSES measure.

#### Analysis

Sample 2 data were used for the purposes of Exploratory Factor Analysis (EFA). Principal Axis Factoring extraction and promax rotation (kappa set at 4) were used to extract factors. Principal Axis Factoring is the most widely used method of factor analysis in the social sciences [22] and oblique rotation methods were suggested because some factors were expected to be correlated [23].

### Results

We began by assessing the suitability of our data for factor analysis. The Kaiser-Meyer-Olkin (KMO) measure of sampling adequacy was .72 and therefore higher than the suggested cut-off point of .60 [24]. Furthermore, Bartlett’s test of sphericity, *χ*^2^ (1176) = 4268.80, *p* < .05 showed that the correlation matrix was not an identity matrix and was appropriate for factor analysis. We therefore proceeded to EFA.

In order to identify the underlying factor structure we considered both Kaiser’s eigenvalues >1 and scree plots of the data [25]. In both cases they yielded 9 factors. However, an eigenvalue of 0.935 for a tenth factor led to the decision to explore both 9 and 10 factor solutions. The research team ultimately decided that the 10 factor solution was conceptually more reasonable and therefore took the 10 factor solution forward to the main study. The 9 factor solution joined perceptions of self in Maths and Science in a single factor whereas the 10 factor solution distinguished between perceptions relating to the three core GCSE subjects. The 10 factor solution explained 63% of the variance in participants’ scores and can be seen in Table 1.

**Table 1:** SENSES—The 10 factor solution and factor correlations.

Factor	Sample item	Number of items	Eigen values	Variance explained (%)	Cronbach’s alpha	Factor correlations								
						2	3	4	5	6	7	8	9	10
1. ENGLISH: perceptions of self and teacher.	My English teacher answered my questions fully and carefully.	9	9.384	19.15	.89	-.17	.20	.18	.12	.24	.19	.42	.26	-.06
2. EFFORT (English. Maths and Science)	I should have worked harder on my English/Maths/Science coursework.	6	4.686	9.56	.92		-.33	-.07	-.22	-.14	.10	-.29	.07	-.02
3. SCIENCE 1: perceptions of self	I was good at Science	5	3.812	7.78	.89			.09	.50	.24	.07	.31	.20	.10
4. MATHS 2: perceptions of teacher	My maths teacher made sure I understood what I needed to do in the course	4	2.979	6.08	.90				.32	.30	.12	.17	-.17	.06
5. MATHS 1: perceptions of self	I was confident that I would get an excellent grade in my maths GCSE	5	2.412	4.92	.88					.18	.13	.29	-.04	.08
6. SCIENCE 2: perceptions of teacher	My science teacher encouraged me to ask questions.	4	1.993	4.07	.85						.12	.21	-.10	-.11
7. PLANS 1: family influence	My plans for after Year 11 were influenced by competitiveness between me and my twin/sibling(s).	5	1.929	3.94	.79							.14	.21	.09
8. PLANS 2: self-confidence	I am confident I can live up to what my parents expect of me.	4	1.450	2.96	.82								.05	.12
9. SOCIAL MEDIA CONNECTIONS	I get a lot of useful information through social media sites.	4	1.312	2.68	.77									.00
10. PLANS 3: work experience	My plans for the future were influenced by interesting work training/experience.	3	.935	1.91	.77									
**TOTAL**		49		63.05	.85									

It is interesting to note that while some of these factors relate to experiences that can relatively easily be classified as ‘environmental’, such as teachers, social media and the influence of family and work experience; others describe aspects of *behaviour* such as effort and confidence that would not typically be considered as environmental influences. However, it is important to note that behavioural discordance in MZ twins must have non-shared environmental origins, that is, if one MZ twin is more confident or hard-working than the other this has to be for environmental reasons, and that such discordant behaviour could have NSE effects. For this reason, we retained both types of factor and both types of item. In some cases it is difficult to draw a clear line between experience and behaviour. For instance, does an individual’s perception or interpretation of an event, such as an interaction with a teacher, represent environment or behaviour? Our focus here was on individual experiences (including perceptions) that can explain NSE variance.

Table 1 also shows a sample item for each factor, the number of items representing each factor, the proportion of variance explained and Cronbach’s alpha. It can be seen that all factors showed good internal consistency with Cronbach’s alphas ranging from .76 to .92. The English (perceptions of self and teacher) factor explained the largest amount of variance (19.15%). The combined variance explained by the split Science and Maths factors was a little lower (11.85% for Science and 11% for Maths). Items relating to Maths and Science did not combine in a single factor as they did for English. Effort during GCSE courses accounted for a similar amount of variance (9.56%). Factors relating to social media and to future plans explained smaller proportions of variance (<4%).

Table 2 shows that factor pattern coefficients suggested all of the items had factor loadings above .40 [25] (See Table 2).

**Table 2:** SENSES–communalities and factor coefficients.

	Factor																				
Items	Com.	ENGLISH		EFFORT		SCIENCE 1		MATHS 2		MATHS 1		SCIENCE 2		PLANS 1		PLANS 2 (SC)		SOC MED		PLANS 3	
						(PS)		(PT)		(PS)		PT)		(F)						(WE)	
		Pat. Str.		Pat. Str.		Pat. Str.		Pat. Str.		Pat. Str.		Pat. Str.		Pat. Str.		Pat. Str.		Pat. Str.		Pat. Str.	
ITEM 1.1	0.80	**0.85**	**0.84**	-0.02	-0.09	-0.24	-0.05	0.14	0.31	-0.02	0.03	0.13	0.31	0.02	0.18	-0.03	0.30	-0.03	0.11	-0.01	-0.09
ITEM 1.2	0.54	**0.77**	**0.72**	-0.02	-0.11	-0.03	0.09	-0.11	0.03	0.02	0.05	-0.04	0.11	0.05	0.16	-0.05	0.25	-0.04	0.19	-0.02	-0.07
ITEM 1.3	0.68	**0.74**	**0.76**	-0.01	-0.22	0.18	0.37	-0.18	-0.01	0.09	0.23	-0.07	0.12	-0.03	0.12	0.09	0.44	-0.01	0.26	0.05	0.04
ITEM 1.4	0.51	**0.71**	**0.66**	-0.07	-0.14	0.04	0.18	-0.13	-0.04	0.05	0.07	-0.08	0.03	-0.01	0.12	-0.14	0.18	0.13	0.34	0.05	0.00
ITEM 1.5	0.75	**0.70**	**0.79**	-0.06	-0.26	0.25	0.42	0.02	0.12	-0.06	0.18	-0.11	0.12	-0.04	0.14	0.10	0.47	0.12	0.36	0.08	0.08
ITEM 1.6	0.62	**0.68**	**0.67**	0.09	0.00	-0.22	-0.06	0.08	0.28	0.04	0.06	0.27	0.41	-0.02	0.12	-0.01	0.25	-0.11	-0.02	-0.07	-0.16
ITEM 1.7	0.58	**0.64**	**0.64**	0.04	-0.03	-0.15	-0.08	0.27	0.39	-0.09	0.01	0.03	0.25	0.06	0.16	0.02	0.24	-0.17	-0.07	-0.12	-0.16
ITEM 1.8	0.47	**0.63**	**0.60**	0.02	-0.04	0.15	0.15	0.08	0.06	-0.22	-0.11	-0.05	0.06	-0.08	0.05	-0.14	0.12	0.18	0.35	0.06	0.00
ITEM 1.9	0.63	**0.63**	**0.68**	-0.03	-0.26	0.26	0.43	-0.13	0.01	0.07	0.25	-0.14	0.06	-0.08	0.07	0.14	0.47	0.02	0.26	0.08	0.10
ITEM 2.1	0.74	-0.08	-0.17	**0.86**	**0.83**	0.06	-0.17	-0.03	-0.02	0.05	-0.09	0.15	0.03	0.07	0.17	-0.02	-0.23	-0.01	0.04	0.04	0.03
ITEM 2.2	0.74	0.15	0.02	**0.85**	**0.83**	-0.09	-0.29	-0.06	-0.04	0.04	-0.15	0.10	-0.01	0.08	0.20	0.03	-0.15	-0.02	0.07	0.07	0.04
ITEM 2.3	0.77	0.10	-0.02	**0.83**	**0.84**	0.10	-0.24	0.00	-0.08	-0.20	-0.32	0.08	-0.02	0.08	0.17	-0.03	-0.24	0.03	0.14	-0.06	-0.09
ITEM 2.4	0.74	-0.20	-0.31	**0.82**	**0.80**	0.00	-0.23	-0.01	-0.11	0.13	-0.10	-0.12	-0.27	-0.15	-0.06	0.11	-0.22	0.11	0.10	0.01	0.03
ITEM 2.5	0.72	0.01	-0.21	**0.77**	**0.81**	0.05	-0.31	0.09	-0.05	-0.04	-0.23	-0.27	-0.35	-0.02	0.01	-0.06	-0.32	-0.06	0.01	0.00	0.01
ITEM 2.6	0.68	-0.08	-0.24	**0.75**	**0.80**	-0.02	-0.34	-0.06	-0.18	-0.09	-0.31	-0.04	-0.21	-0.06	-0.01	0.02	-0.30	0.05	0.08	-0.01	-0.03
ITEM 3.1	0.79	0.14	0.31	-0.03	-0.34	**0.81**	**0.87**	0.02	0.17	0.07	0.51	-0.01	0.27	0.06	0.13	0.01	0.35	-0.10	0.11	-0.04	0.04
ITEM 3.2	0.55	-0.10	0.06	-0.04	-0.22	**0.78**	**0.68**	0.20	0.21	-0.20	0.24	0.11	0.29	0.03	0.05	-0.13	0.09	0.00	0.09	-0.03	0.02
ITEM 3.3	0.56	-0.06	0.07	0.07	-0.17	**0.72**	**0.73**	-0.03	0.08	0.12	0.44	0.05	0.21	0.03	0.08	-0.05	0.17	-0.03	0.10	0.01	0.08
ITEM 3.4	0.65	0.06	0.22	0.04	-0.26	**0.66**	**0.77**	-0.07	0.12	0.22	0.54	0.08	0.29	0.01	0.09	0.03	0.32	-0.07	0.09	-0.04	0.03
ITEM 3.5	0.69	0.10	0.30	0.05	-0.26	**0.61**	**0.78**	-0.11	0.08	0.23	0.54	0.15	0.34	-0.04	0.08	0.08	0.36	0.05	0.19	-0.07	-0.01
ITEM 4.1	0.84	-0.05	0.14	-0.06	-0.11	-0.01	0.12	**0.92**	**0.91**	0.03	0.33	0.05	0.30	-0.04	0.07	-0.02	0.15	0.07	-0.12	0.02	0.07
ITEM 4.2	0.79	0.00	0.18	-0.01	-0.12	0.05	0.20	**0.84**	**0.87**	0.12	0.40	0.03	0.29	-0.11	0.03	-0.01	0.19	0.08	-0.09	0.05	0.09
ITEM 4.3	0.71	0.03	0.23	0.01	-0.08	0.05	0.14	**0.81**	**0.83**	-0.01	0.31	-0.06	0.23	0.10	0.21	0.11	0.27	-0.01	-0.10	-0.02	0.05
ITEM 4.4	0.47	-0.05	0.05	-0.03	-0.11	-0.03	0.14	**0.56**	**0.63**	0.25	0.41	-0.02	0.16	-0.07	0.01	0.01	0.15	-0.04	-0.18	0.12	0.17
ITEM 5.1	0.76	-0.05	0.08	0.01	-0.22	0.09	0.47	0.11	0.35	**0.79**	**0.86**	-0.04	0.14	-0.11	0.01	0.06	0.29	-0.01	-0.06	0.01	0.09
ITEM 5.2	0.63	0.00	0.06	-0.01	-0.17	0.01	0.38	0.00	0.28	**0.78**	**0.77**	0.12	0.25	0.02	0.11	-0.13	0.13	-0.06	-0.10	0.02	0.06
ITEM 5.3	0.66	0.01	0.22	-0.12	-0.30	0.08	0.51	0.05	0.28	**0.66**	**0.77**	0.02	0.20	0.06	0.19	0.07	0.35	0.14	0.13	0.01	0.08
ITEM 5.4	0.69	-0.06	0.16	-0.08	-0.28	0.20	0.51	0.28	0.46	**0.52**	**0.75**	-0.14	0.13	0.15	0.24	0.13	0.38	-0.01	0.00	-0.06	0.07
ITEM 5.5	0.55	-0.06	0.00	0.17	0.01	0.31	0.42	0.34	0.46	**0.44**	**0.61**	-0.05	0.15	0.01	0.10	-0.15	0.05	-0.07	-0.08	-0.01	0.06
ITEM 6.1	0.74	-0.07	0.15	-0.06	-0.18	-0.02	0.21	0.02	0.27	0.00	0.16	**0.86**	**0.85**	-0.06	0.04	0.05	0.20	-0.01	-0.14	0.05	-0.04
ITEM 6.2	0.71	0.11	0.34	0.07	-0.12	0.15	0.38	0.02	0.27	0.07	0.27	**0.75**	**0.80**	-0.09	0.08	0.02	0.26	0.10	0.06	0.01	-0.07
ITEM 6.3	0.54	-0.18	-0.04	-0.01	-0.14	0.27	0.39	-0.19	0.01	0.04	0.20	**0.67**	**0.62**	-0.01	0.04	-0.06	0.08	-0.02	-0.05	0.14	0.09
ITEM 6.4	0.60	0.09	0.36	0.00	-0.15	0.10	0.30	0.17	0.38	-0.04	0.22	**0.60**	**0.72**	0.07	0.20	0.10	0.32	0.05	0.02	-0.07	-0.11
ITEM 7.1	0.82	-0.12	0.10	-0.06	0.02	0.03	0.12	-0.05	0.08	0.03	0.17	0.05	0.15	**0.91**	**0.89**	0.05	0.16	0.00	0.16	-0.02	0.08
ITEM 7.2	0.70	-0.06	0.10	-0.02	0.09	0.01	0.10	-0.06	0.01	0.07	0.13	0.04	0.08	**0.80**	**0.81**	-0.13	0.00	0.15	0.30	0.04	0.10
ITEM 7.3	0.36	0.06	0.11	0.06	0.10	0.07	0.10	0.02	0.09	0.10	0.17	-0.12	-0.03	**0.56**	**0.57**	-0.09	0.03	-0.05	0.10	0.07	0.13
ITEM 7.4	0.36	0.04	0.19	0.06	0.07	0.11	0.06	-0.05	-0.02	-0.20	-0.06	-0.02	0.07	**0.55**	**0.54**	0.16	0.19	-0.04	0.14	-0.13	-0.07
ITEM 7.5	0.39	0.08	0.07	0.04	0.16	-0.10	-0.12	0.06	0.05	-0.04	-0.04	-0.16	-0.15	**0.52**	**0.52**	-0.10	-0.04	0.01	0.12	0.23	0.27
ITEM 8.1	0.83	-0.01	0.35	0.08	-0.18	-0.15	0.19	-0.05	0.16	0.17	0.34	0.04	0.21	-0.01	0.12	**0.92**	**0.89**	-0.05	-0.04	-0.05	0.06
ITEM 8.2	0.77	0.05	0.40	-0.02	-0.25	-0.15	0.27	-0.06	0.16	0.32	0.47	-0.02	0.16	0.08	0.23	**0.75**	**0.83**	0.06	0.09	-0.01	0.09
ITEM 8.3	0.51	-0.03	0.27	0.03	-0.18	0.05	0.18	0.15	0.23	-0.18	0.10	0.04	0.19	-0.05	0.05	**0.71**	**0.68**	-0.03	-0.03	0.04	0.12
ITEM 8.4	0.53	-0.15	0.12	-0.09	-0.24	0.12	0.22	0.01	0.02	-0.26	0.00	0.02	0.08	-0.08	-0.02	**0.62**	**0.57**	0.06	0.06	0.17	0.24
ITEM 9.1	0.43	0.06	0.23	-0.01	0.05	-0.05	0.16	0.02	-0.04	0.15	0.09	0.13	0.06	0.03	0.19	-0.18	-0.06	**0.71**	**0.69**	-0.06	-0.08
ITEM 9.2	0.57	0.01	0.26	0.04	0.07	0.04	0.11	0.07	-0.09	-0.24	-0.17	-0.01	-0.05	0.05	0.20	0.19	0.17	**0.68**	**0.71**	-0.04	-0.03
ITEM 9.3	0.50	0.00	0.22	0.05	0.12	-0.10	0.02	0.06	-0.07	-0.11	-0.12	0.01	-0.06	0.10	0.24	0.12	0.10	**0.67**	**0.67**	-0.02	-0.01
ITEM 9.4	0.41	0.02	0.14	0.02	0.07	-0.08	0.09	0.00	-0.10	0.14	0.04	0.00	-0.09	-0.08	0.07	-0.09	-0.04	**0.65**	**0.62**	0.01	-0.01
ITEM 10.1	0.68	-0.01	-0.02	-0.06	-0.11	0.05	0.15	0.05	0.11	-0.04	0.10	0.01	-0.04	-0.01	0.07	0.06	0.18	-0.04	-0.04	**0.80**	**0.81**
ITEM 10.2	0.59	0.10	0.04	0.10	0.13	-0.24	-0.08	0.03	0.10	0.18	0.11	0.03	-0.08	0.03	0.16	-0.05	0.05	0.10	0.07	**0.73**	**0.71**
ITEM 10.3	0.54	0.01	0.04	0.04	-0.03	0.05	0.09	0.07	0.17	-0.14	0.04	0.13	0.13	0.09	0.15	0.15	0.24	-0.19	-0.16	**0.65**	**0.66**

*Note*. Com.: Communalities, Pat.: Values obtained from Pattern Matrix, Str.: Values obtained from Structure Matrix. SCIENCE 1 (PS) and MATHS 1 (PS) refer to perceptions of self in these subjects. PLANS 1 (F) refers to family influence on plans; PLANS 2 (SC) refers to self-confidence about plans for the future; and PLANS 3 (WE) refers to the influence of work experience on plans for the future. SOCMED refers to participants’ social media use.

The 49th item (Item 5.5)–*I was interested in what we were studying in maths*–was found to have close loadings (differences between primary and alternative factor loadings were less than .20) on 3 different factors (.44 on maths self-perception but also .34 on perceptions of maths teacher and .31 on science self-perception). However, it was noted that the equivalent item for English and Science loaded only on the appropriate factors i.e. perceptions of self in English and Science. In order to maintain consistency of items across three subject domains it was decided to take the 49th item forward to Study 2 for further testing. It has been suggested that the consequences of over-factoring are usually less marked than the consequences of under-factoring [18] and this approach allowed us to test the items and the factor structure with a larger sample, yielding more trustworthy results. Furthermore, it is generally agreed that substantive considerations should be taken into account alongside statistical considerations in EFA [26].

### Discussion

The 10 factors yielded by our multi-stage process were conceptually reasonable, representative of the qualitative data gathered in an earlier phase of this project and they showed good internal reliability. The scales showed both convergent item validity (high factor loadings on a relevant scale) and, with one exception, divergent item validity (low factor loadings on other scales).

The major limitation of this pilot study was that the ratio of items to participants was too great (82 items for PCA and 117 participants i.e. one twin per pair in each sample). However, subsequent analysis found that our data were suitable for factor analysis. A further issue to consider in Study 2, where this problem was eliminated, is that the decision to proceed with a 10 factor rather than a 9 factor model was made on conceptual rather than statistical grounds. We need to ask whether the 49-item, 10 factor structure is confirmed. These issues are revisited in Study 2 and in the *General Discussion*.

## Study 2

### Aims

■ Conduct Confirmatory Factor Analysis (CFA) to evaluate the construct validity of SENSES.
■ Explore external validity via correlations with GCSE performance and self-reported life satisfaction.

### Method

#### Participants

Participants for Study 2 were also drawn from the Twins’ Early Development Study (TEDS) [16, 17]. We invited twins in 2165 families to participate and received SENSES and life-satisfaction data from *n* = 926 families (53% MZ). Twins were provided with a detailed information sheet and questionnaire completion implied consent. This approach was approved by our institutional ethics committee. In 908 cases we received data from both twins in the pair and in 18 cases, only from one. Data were gathered, therefore, from *n* = 1834 individuals (Mean age = 18.4; Range = 17 to 19; 61.6% female). Of these, *n* = 1672 participants had previously provided us with academic achievement data and, in all but 3 cases, this included General Certificate of Secondary Education (GCSE) data. In the remaining three cases alternative examinations to GCSE were taken. The sample was not fully representative of the UK population, or of the original TEDS sample. The relatively increased proportion of girls (from close to 50% at first contact) is broadly representative of TEDS data at age 16, but not of the UK population. This discrepancy may be the result of a greater willingness to engage with data collection among girls than boys at this age. Furthermore, standardized SES was higher in this sample than in the full TEDS sample (*M* = 0.31), and, more surprisingly, standardized g scores (measured at age 12) were slightly lower (*M* = -0.12). These discrepancies may be due to sample selection effects.

#### Measures

The 49 item SENSES measure developed in Study 1 was administered to Study 2 participants (*n* = 926 twin pairs). Data had previously been gathered on GCSE results and were also gathered on self-perceived life satisfaction using a well-validated five item measure of global life satisfaction [27]. Items included ‘In most ways my life is close to my ideal’ and ‘If I could live my life over, I would change almost nothing’ and used a 5 point response scale from 1 = Strongly disagree through to 5 = Strongly agree. In the current study α = 0.86.

#### Procedure

Questionnaires were posted to twin pairs along with an information sheet and separate envelopes for individual questionnaires so that twins could retain their privacy. Because the twins had already completed their GCSEs they were specifically asked to think back to Year 10 and 11 (when they were taking GCSE courses) when responding to items. Twins who returned completed questionnaires received a £5 gift voucher each and were entered into a prize draw with a chance of winning a pair of iPad minis.

#### Analysis

Confirmatory Factor Analysis (CFA) was conducted using the maximum likelihood estimation method in LISREL 8.80 [28] with SIMPLIS command language. One twin per pair was randomly selected for CFA and invariance analysis was conducted with this sample and the co-twin sample. Correlations between SENSES and our measures of achievement and life satisfaction were conducted. All analyses were replicated with the co-twin sample.

### Results

CFA found an acceptable model fit to the data (^2^_(1082)_ = 4992.29, *p* < .05; CFI = .93; NFI = .92; SRMR = .053 RMSEA = .071; 90% CI = .070, .073) [29,30]. All items loaded significantly on their intended factors and standardized parameter estimates (Lambda X) ranged from .49 to .92 (See Table 3). Here we want to emphasize that the 49^th^ item (*i*.*e*. *I was interested in what we were studying in Maths*), which we retained in the interests of retaining consistency across domains, had a sufficiently high factor loading of. 77. Therefore, this analysis with a larger sample supported our decision to retain this item.

**Table 3:** SENSES—Standardized parameter estimates (Lambda-X).

Item	Factor	λ
My English teacher(s) made sure I understood what I needed to do in the course	ENGLISH	.79**
My English teacher(s) was excellent	ENGLISH	.81**
I was confident I could live up to what my English teacher(s) expected.	ENGLISH	.74**
I was good at this subject (English)	ENGLISH	.61**
I was confident I could master the skills we learned in English	ENGLISH	.70**
My English teacher(s) answered my questions fully and carefully.	ENGLISH	.80**
My English teacher(s) encouraged me to ask questions.	ENGLISH	.69**
I was confident that I would get an excellent grade in my English GCSE(s).	ENGLISH	.68**
I was interested in what we were studying in English.	ENGLISH	.60**
I should have worked harder on my English coursework.	EFFORT	.72**
I should have revised harder for my English exams.	EFFORT	.73**
My Maths teacher(s) answered my questions fully and carefully.	MATHS 2 (PT)	.88**
My Maths teacher(s) made sure I understood what I needed to do in the course.	MATHS 2 (PT)	.92**
My Maths teacher(s) encouraged me to ask questions.	MATHS 2 (PT)	.83**
My Maths teacher(s) was excellent.	MATHS 2 (PT)	.85**
I was confident that I would get an excellent grade in my Maths GCSE(s).	MATHS 1 (PS)	.91**
I was good at this subject (Maths)	MATHS 1 (PS)	.87**
I was confident I could live up to what my Maths teacher(s) expected.	MATHS 1 (PS)	.89**
I was interested in what we were studying in Maths.	MATHS 1 (PS)	.77**
I was confident I could master the skills we learned in Maths.	MATHS 1 (PS)	.90**
I should have revised harder for my Maths exams.	EFFORT	.75**
I should have worked harder on my Maths coursework.	EFFORT	.72**
My Science teacher(s) answered my questions fully and carefully.	SCIENCE 2 (PT)	.89**
My Science teacher(s) made sure I understood what I needed to do in the course.	SCIENCE 2 (PT)	.91**
My Science teacher(s) was excellent.	SCIENCE 2 (PT)	.86**
My Science teacher(s) encouraged me to ask questions.	SCIENCE 2 (PT)	.80**
I was confident I could master the skills we learned in Science.	SCIENCE 1 (PS)	.87**
I was interested in what we were studying in Science.	SCIENCE 1 (PS)	.73**
I was good at this subject (Science).	SCIENCE 1 (PS)	.88**
I was confident that I would get an excellent grade in my Science GCSE(s).	SCIENCE 1 (PS)	.92**
I was confident I could live up to what my Science teacher(s) expected.	SCIENCE 1 (PS)	.88**
I should have revised harder for my Science exams.	EFFORT	.78**
I should have worked harder on my Science coursework.	EFFORT	.81**
My plans for after Year 11 were influenced by my father’s career choice or life experience.	PLANS 1 (F)	.69**
My plans for after Year 11 were influenced by my mother’s career choice or life experience.	PLANS 1 (F)	.64**
My plans for after Year 11 were influenced by an adult role model or mentor.	PLANS 1 (F)	.75**
My plans for after Year 11 were influenced by my twin/sibling’s plans–I want a similar future.	PLANS 1 (F)	.56**
My plans for after Year 11 were influenced by competitiveness between me and my twin/sibling(s).	PLANS 1 (F)	.49**
My plans for after Year 11 were influenced by volunteering experiences.	PLANS 3 (WE)	.63**
My plans for after Year 11 were influenced by part-time job experiences.	PLANS 3 (WE)	.65**
My plans for after Year 11 were influenced by interesting work training/experience.	PLANS 3 (WE)	.78**
When using social media sites, I feel connected with others.	SOC MED	.64**
My social media posts are well received (e.g., Like, Favourite, RT).	SOC MED	.77**
I have a wide social media network (e.g. Facebook friends).	SOC MED	.76**
I get a lot of useful information through social media sites.	SOC MED	.58**
I am confident I can live up to what my parents expect of me.	PLANS 2 (SC)	.84**
I am confident I can live up to what my teachers expect of me.	PLANS 2 (SC)	.89**
I can confident I can live up to what I expect of myself.	PLANS 2 (SC)	.67**
I have a clear plan for what I hope to do next.	PLANS 2 (SC)	.51**

** p < .01

SCIENCE 1 (PS) and MATHS 1 (PS) refer to perceptions of self in these subjects. PLANS 1 (F) refers to family influence on plans; PLANS 2 (SC) refers to self-confidence about plans for the future; and PLANS 3 (WE) refers to the influence of work experience on plans for the future. SOCMED refers to participants’ social media use.

We looked at correlations between our 10 factors and found an average correlation of r = 0.16 (range = .01 to .69). This suggested that the most factors showed low or no levels of correlation and can, therefore, be reasonably considered to be measuring different things (See Table 4).

**Table 4:** Factor correlations (Phi estimates).

	ENGLISH	EFFORT	SCIENCE 1 (PS)	MATHS 2 (PT)	SCIENCE 2 (PT)	PLANS 1 (F)	PLANS 2 (SC)	MATHS 1 (PS)	SOCMED	PLANS 1 (WE)
ENGLISH										
EFFORT	-.19*									
SCI 1 (PS)	.19*	-.31*								
MATHS 2 (PT)	.15*	-.14*	.23*							
SCI 2 (PT)	.29*	-.17*	.69*	.21*						
PLANS 1 (F)	.04	.13*	.09*	.06	.11*					
PLANS 2 (SC)	.25*	-.16*	.24*	.15*	.19*	.08*				
MATHS 1 (PS)	.06	-.25*	.46*	.64*	.24*	.11*	.22*			
SOCMED	.13*	.08*	-.02	.07	.03	.10*	.22*	.01		
PLANS3 (WE)	-.03	.12*	.03	.02	.05	.38*	.11*	.01	.19*	

*: significant at 0.05 level.

SCIENCE 1 (PS) and MATHS 1 (PS) refer to perceptions of self in these subjects. PLANS 1 (F) refers to family influence on plans; PLANS 2 (SC) refers to self-confidence about plans for the future; and PLANS 3 (WE) refers to the influence of work experience on plans for the future. SOCMED refers to participants’ social media use.

Three correlations were exceptions to this pattern. The correlation between SCIENCE 1 (Perceptions of Self) and SCIENCE 2 (Perceptions of Teacher) was r = .69, p < .05. A similar pattern was also observed for Maths in that MATHS 1 and MATHS 2 correlated r = .64, p < .01. Finally, self-perceptions in science (SCIENCE 1) also correlated r = 0.46, p < .05 with MATHS 2 (perceptions of Maths teacher). These correlations and their implications for the SENSES measure are discussed later.

Table 5 shows means, standard deviations and reliability coefficients for the 10 factors.

**Table 5:** Factor means, standard deviations and reliabilities.

Factors	Mean	SD	Cronbach Alpha (α)
ENGLISH	3.53	.82	.90
EFFORT	2.53	1.01	.89
SCIENCE 1 (PS)	3.59	.99	.93
MATHS 2 (PT)	3.39	1.11	.94
MATHS 1 (PS)	3.73	1.03	.93
SCIENCE 2 (PT)	3.65	.94	.92
PLANS(F)	1.79	.78	.76
PLANS(SC)	3.66	.88	.81
SOCMED	3.38	.83	.78
PLANS(WE)	1.99	.97	.72

As in Study 1 all 10 factors showed good levels of internal reliability with Cronbach’s alphas ranging from .72 to .94. Mean scores (based on 5 point response formats with 1 = not at all true and 5 = very true) were highest for English (M = 3.53), Maths (MATHS 1 M = 3.73; MATHS 2 M = 3.39), Science (SCIENCE 1 M = 3.59; SCIENCE 2 M = 3.65), Self-confidence about future plans (PLANS 2 M = 3.66) and Social Media (M = 3.38). They were lowest for the influence of family (PLANS 1 M = 1.79) and work experience (PLANS 3 M = 1.99) and were mid-range for effort (M = 2.53). Standard deviations ranged from a low of .78 for PLANS 1 (family influence) to 1.11 for MATHS 2 (perceptions of Maths teachers).

We also looked at factorial invariance across the two samples (one twin per pair in each sample group) in order to cross-validate the 10 factor model. This was achieved by conducting five multi-group CFA models. Firstly, a baseline model was tested in order to examine whether both samples conceptualised the constructs in a similar manner. In the remaining models factor loadings, factor variance, factor covariance and variance of error terms were constrained to be equal across the two samples and invariance between the models was compared (See Table 6)

**Table 6:** Tests for invariance of NSE model across two groups: Summary of goodness-of-fit statistics.

Model	*Χ*^*2*^	*df*	RMSEA	IFI	NNFI	CFI	*Model comparison*	*ΔΧ*^*2*^	Δ*df*	ΔCFI
Model 1:Baseline	10346.201	2164	.0730	.929	.923	.929	-	-	-	-
Model 2: Factor loadings invariant	10394.062	2203	.0726	.929	.924	.929	2 vs 1	47.861^ns^	39	.000
Model 3: Factor loadings and factor variances invariant	10398.781	2213	.0725	.929	.925	.929	3 vs 2	4.719^ns^	10	.000
Model 4: Factor loadings, factor variances and factor covariances invariant	10435.289	2258	.0717	.929	.926	.929	4 vs 3	36.508^ns^	45	.000
Model 5:Factor loadings, factor variances, factor covariances and variance of error terms invariant	10502.441	2307	.0710	.929	.928	.929	5 vs 4	67.152^ns^	49	.000

^ns^: nonsignificant

Comparisons of each model found that chi-squared differences were non-significant. Furthermore, ΔCFI between constrained and unconstrained models were less than .01, as suggested by Cheung & Rensvold [31]. In summary, invariance testing supported the factorial invariance of SENSES across our two related samples.

#### External validity

In order to assess the external validity of the SENSES measure we looked at correlations between the 10 factors and both GCSE achievement in English, Maths and Science and self-reported life satisfaction (See Table 7).

**Table 7:** External validity analysis: Correlations between SENSES and GCSE achievement and life satisfaction.

Factor	English	Maths	Science	Life Satisfaction
ENGLISH: Perceptions of Self and Teacher	0.39** (N = 832)	0.11** (N = 825)	0.10** (N = 775)	0.21** (N = 905)
EFFORT: English, Maths and Science	0.39** (N = 832)	0.38** (N = 825)	0.41** (N = 775)	0.15** (N = 905)
SCIENCE 1: Perceptions of Self	0.21** (N = 832)	0.32** (N = 825)	0.49** (N = 775)	0.11** (N = 905)
MATHS 2: Perceptions of Teacher	0.09** (N = 832)	0.31** (N = 825)	0.19** (N = 775)	0.19** (N = 905)
MATHS 1: Perceptions of Self	0.14** (N = 832)	0.53** (N = 825)	0.38** (N = 775)	0.15** (N = 905)
SCIENCE 2: Perceptions of Teacher	0.11** (N = 832)	0.16** (N = 825)	0.25** (N = 775)	0.14** (N = 905)
PLANS 1: Family influence	0.06 (N = 832)	0.05 (N = 825)	0.06 (N = 775)	0.05 (N = 905)
PLANS 2: Self-confidence	-0.05 (N = 832)	-0.07* (N = 825)	-0.08 (N = 775)	0.48** (N = 905)
SOCIAL MEDIA CONNECTIONS	-0.03 (N = 832)	-0.10** (N = 825)	-0.13** (N = 775)	0.13** (N = 905)
PLANS 3: Work experience	-0.04 (N = 832)	-0.07 (N = 825)	-0.02* (N = 775)	0.08* (N = 905)

*p< .05

**p< .01.

#### GCSE achievement

In general, the domain specific factors (Perceptions of Self and Teacher in English, Maths and Science) were significant correlates of GCSE achievement in English, Maths and Science respectively. The ENGLISH factor correlated r = .39, p < .001 with GCSE English but only r = .11, p < .01 with Maths and r = .10, p < .01 with Science. Likewise, SCIENCE factors correlated more strongly with Science achievement than with English or Maths achievement, and MATHS factors correlated more strongly with Maths achievement than with English or Science. Perceptions of self in Science correlated r = .49, p < .001 with Science GCSE, r = .32, p < .001 with Maths and r = .21, p < .001 with English. Perceptions of Science teachers correlated r = .25, p < .001 with Science GCSE, r = .16, p < .001 with Maths and r = .11, p < .001 with English. Perceptions of self in Maths correlated r = .31, p < .001 with Maths GCSE, r = .19, p < .001 with Science and r = .09, p < .01 with English. Perceptions of Maths teachers correlated r = .53, p < .001 with Maths GCSE, r = .38, p < .001 with Science and r = .14, p < .001 with English. Effort correlated at a similar level with all three domains (average r = .39, p < .001).

However, the remaining four factors yielded few significant correlations with GCSE achievement and those that did achieve statistical significance ranged from r = -0.07, p < .05 for the correlation between self-confidence about the future (PLANS 2) and Maths achievement, and r = -.11, p < .01 for the correlation between Social Media and Science achievement.

#### Life satisfaction

Correlations between the SENSES factors and our measure of life satisfaction were, with one exception statistically significant but mainly weak, ranging from r = .05 (NS) for PLANS 1 (family influence) and r = .08, p < .05 for PLANS 3 (work experience) through to a moderate correlation of r = .48, p < .001 for PLANS 2 (self-confidence about the future). The average correlation was r = .13.

### Discussion

Study 2 did not have the principal limitations of Study 1 in that we administered a questionnaire with fewer items (49 compared with 82) to a larger sample (*n* = 926 twin pairs compared with *n* = 117 twin pairs). It was therefore pleasing to note that CFA confirmed the factor structure that emerged from Study 1, and justified our decisions to use a 10 factor structure (rather than a 9 factor structure) and to retain 49 rather than 48 items. It was also seen that all 10 of the SENSES factors retained good levels of internal reliability. Furthermore, invariance testing cross-validated the model across our two related samples.

One area of concern was that although most factors correlated at the level of r</ = ~.3 there were three exceptions. The two Maths factors correlated r = .64; the two Science factors correlated r = .69 and self-perceptions in Science correlated r = .46 with perceptions of teacher in Maths. We also know that the same variables in relation to English did not split into two separate factors and the pattern is therefore inconsistent across the three GCSE subjects. This raises the possibility that either English should be split into two factors or that the currently separate but correlated Maths factors should be joined (also true for Science). It should remain a consideration that in the 9 factor structure suggested by EFA self-perceptions in Maths and Science were found to cluster on a single factor. This issue can only be satisfactorily resolved through further testing in different samples. We will not be able to reasonably claim the SENSES instrument is robust until we have tested it in more populations. However, in the meantime, it can be considered positive that ENGLISH factors correlated most strongly with English achievement, MATHS factors with Maths achievement and SCIENCE factors with Science achievement, suggesting external validity for the existing sub-scales.

## General discussion

The psychometric properties of the SENSES measure appear promising and indicate that it, or sub-scales from it, can make a useful contribution to research. However, some issues remain to be resolved in future research with different samples. Only by conducting further validation research will we gain confidence that we have identified the optimal factor structure. As the measure stands, domain specific factors show moderate correlations with domain specific GCSE achievement; and our measure of self-confidence about the future (PLANS 2) shows a moderate association with self-reported life satisfaction in late adolescence.

Four of the SENSES factors did not correlate with either GCSE achievement or life satisfaction. However, items were developed on the basis of discordance in a wider range of educationally relevant traits than this, and it is possible that these four factors could correlate with, for example, measures of occupational success, vocational interests or peer relationships. When undertaking further validation work with the SENSES measure it will be important to explore relationships with other variables.

It is important to note that some of the SENSES factors target traits such as effort or beliefs such as self-confidence about the future rather than environments *per se*. The suggestion from the qualitative data is that these factors differ between monozygotic twins and lead to non-shared outcomes. However, this discordance remains to be explained by differences in experience.

### Limitations

We have mentioned the limitation that while we gathered data on a wide range of traits in order to develop the SENSES measure we were only able to test it in relation to GCSE achievement and self-reported global life satisfaction. It would be interesting to explore relationships between SENSES factors and other variables, not measured in the current study, such as personality, peer relationships and mental health status. This can be achieved as we continue to test the validity of the SENSES measure as a whole, and individual sub-scales from it. Our study is also limited by a cross-sectional design that cannot speak to direction of effects or identify reverse causation. Furthermore, we have not yet established whether SENSES can do what it aims to do, that is, to explain NSE variance in outcomes including GCSE achievement and life satisfaction.

A particularly major limitation of this research is that it relied on retrospective data. Specifically, participants already knew their GCSE results when they provided data about their learning experiences during the GCSE course. This may have coloured their view of the GCSE experience in either positive or negative ways. Testing the SENSES measure with a sample of 14–16 year old UK pupils could address this concern.

### Future research

The top priority for future research has to be reliability and validity testing of the SENSES measure in different populations. This will help us to address remaining concerns about whether we have identified the optimal factor structure. Beyond that, longitudinal work is needed if we are to begin to be able to understand the direction of any effects and to test for reverse causation i.e. the possibility that discordant GCSE results or life satisfaction are the precursor to discordant experiences, rather than the other way around. Finally, it will be important to assess whether SENSES factors can actually explain NSE variance in educationally relevant variables, and whether associations are mediated by genetic, shared or non-shared environmental effects (using multivariate twin analyses) and this research is already underway using the current sample.

## Supporting information

S1 File SENSES questionnaire. SENSES Questionnaire.
(DOCX)

S1 Table Study 1 Covariance Matrix. Study 1 Covariance Matrix.
(XLSX)

S2 Table Study 2 Covariance Matrix. Study 2 Covariance Matrix.
(XLSX)
