# Supplementary material for: Developing SENSES: Student experience of non-shared environment scales
Source: PLoS One. 2018 Sep 6;13(9):e0202543. doi: 10.1371/journal.pone.0202543 (PMC6126800; doi:10.1371/journal.pone.0202543)
Supplement: S1 File — SENSES Questionnaire. (DOCX) [file pone.0202543.s001.docx]

**
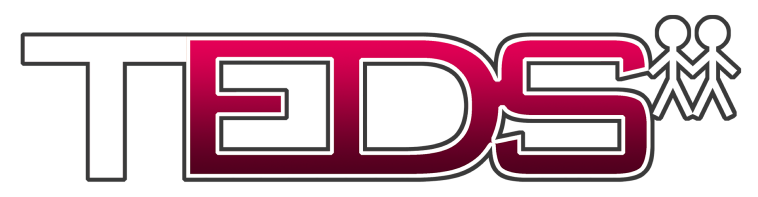
**

**Twin ID:** <twin ID>

**Name: <Twin name>**

**S1 File: SENSES Questionnaire**

**The following statements are about your experiences during your GCSEs. Thinking back to Years 10 and 11 please read each statement and place an X in the box that describes how true it was for you.**

|  | **Not at all true** |  | **Somewhat true** |  | **Very true** |
| --- | --- | --- | --- | --- | --- |
| My English teacher(s) made sure I understood what I needed to do in the course | 🞎 | 🞎 | 🞎 | 🞎 | 🞎 |
| My English teacher(s) was excellent | 🞎 | 🞎 | 🞎 | 🞎 | 🞎 |
| I felt confident I could live up to what my English teacher(s) expected | 🞎 | 🞎 | 🞎 | 🞎 | 🞎 |
| I was good at English | 🞎 | 🞎 | 🞎 | 🞎 | 🞎 |
| I felt confident I could master the skills we learned in English | 🞎 | 🞎 | 🞎 | 🞎 | 🞎 |
| My English teacher(s) answered my questions fully and carefully | 🞎 | 🞎 | 🞎 | 🞎 | 🞎 |
| My English teacher(s) encouraged me to ask questions | 🞎 | 🞎 | 🞎 | 🞎 | 🞎 |
| I felt confident that I would get an excellent grade in my English GCSE(s) | 🞎 | 🞎 | 🞎 | 🞎 | 🞎 |
| I felt interested in what we were studying in English | 🞎 | 🞎 | 🞎 | 🞎 | 🞎 |
| I should have worked harder on my English coursework. | 🞎 | 🞎 | 🞎 | 🞎 | 🞎 |
| I should have revised harder for my English exams | 🞎 | 🞎 | 🞎 | 🞎 | 🞎 |
|  | **Not at all true** |  | **Somewhat true** |  | **Very true** |
| My Maths teacher(s) answered my questions fully and carefully | 🞎 | 🞎 | 🞎 | 🞎 | 🞎 |
| My Maths teacher(s) made sure I understood what I needed to do in the course | 🞎 | 🞎 | 🞎 | 🞎 | 🞎 |
| My Maths teacher(s) encouraged me to ask questions | 🞎 | 🞎 | 🞎 | 🞎 | 🞎 |
| My Maths teacher(s) was excellent | 🞎 | 🞎 | 🞎 | 🞎 | 🞎 |
| I felt confident that I would get an excellent grade in my Maths GCSE(s) | 🞎 | 🞎 | 🞎 | 🞎 | 🞎 |
| I was good at Maths | 🞎 | 🞎 | 🞎 | 🞎 | 🞎 |
| I felt confident I could live up to what my Maths teacher(s) expected | 🞎 | 🞎 | 🞎 | 🞎 | 🞎 |
| I felt interested in what we were studying in Maths | 🞎 | 🞎 | 🞎 | 🞎 | 🞎 |
| I felt confident I could master the skills we learned in Maths | 🞎 | 🞎 | 🞎 | 🞎 | 🞎 |
| I should have revised harder for my Maths exams | 🞎 | 🞎 | 🞎 | 🞎 | 🞎 |
| I should have worked harder on my Maths coursework | 🞎 | 🞎 | 🞎 | 🞎 | 🞎 |
| My Science teacher(s) answered my questions fully and carefully | 🞎 | 🞎 | 🞎 | 🞎 | 🞎 |
| My Science teacher(s) made sure I understood what I needed to do in the course | 🞎 | 🞎 | 🞎 | 🞎 | 🞎 |
| My Science teacher(s) was excellent | 🞎 | 🞎 | 🞎 | 🞎 | 🞎 |
| My Science teacher(s) encouraged me to ask questions | 🞎 | 🞎 | 🞎 | 🞎 | 🞎 |
| I felt confident I could master the skills we learned in Science | 🞎 | 🞎 | 🞎 | 🞎 | 🞎 |
| I felt interested in what we were studying in Science | 🞎 | 🞎 | 🞎 | 🞎 | 🞎 |
| I was good at Science | 🞎 | 🞎 | 🞎 | 🞎 | 🞎 |
| I felt confident that I would get an excellent grade in my Science GCSE(s) | 🞎 | 🞎 | 🞎 | 🞎 | 🞎 |
| I felt confident I could live up to what my Science teacher(s) expected | 🞎 | 🞎 | 🞎 | 🞎 | 🞎 |
| I should have revised harder for my Science exams | 🞎 | 🞎 | 🞎 | 🞎 | 🞎 |
| I should have worked harder on my Science coursework | 🞎 | 🞎 | 🞎 | 🞎 | 🞎 |
| My plans for after Year 11 were influenced by my father’s career choice or life experience | 🞎 | 🞎 | 🞎 | 🞎 | 🞎 |
| My plans for after Year 11 were influenced by an adult role model or mentor | 🞎 | 🞎 | 🞎 | 🞎 | 🞎 |
| My plans for after Year 11 were influenced by my mother’s career choice or life experience | 🞎 | 🞎 | 🞎 | 🞎 | 🞎 |
| My plans for after Year 11 were influenced by my twin (or other sibling)’s plans–I want a similar future | 🞎 | 🞎 | 🞎 | 🞎 | 🞎 |
|  | **Not at all true** |  | **Somewhat true** |  | **Very true** |
| My plans for after Year 11 were influenced by competitiveness between me and my twin (or another sibling) | 🞎 | 🞎 | 🞎 | 🞎 | 🞎 |
| My plans for after Year 11 were influenced by volunteering experiences | 🞎 | 🞎 | 🞎 | 🞎 | 🞎 |
| My plans for after Year 11 were influenced by part-time job experiences | 🞎 | 🞎 | 🞎 | 🞎 | 🞎 |
| My plans for after Year 11 were influenced by interesting work training/experience | 🞎 | 🞎 | 🞎 | 🞎 | 🞎 |

**We are interested in how you use social media. How true are each of these statements for you?**

|  | **Not at all true** |  | **Somewhat true** |  | **Very true** |
| --- | --- | --- | --- | --- | --- |
| When using social media sites, I feel connected with others | 🞎 | 🞎 | 🞎 | 🞎 | 🞎 |
| My social media posts are well received (e.g., Like, Favourite, RT) | 🞎 | 🞎 | 🞎 | 🞎 | 🞎 |
| I have a wide social media network (e.g. Facebook friends) | 🞎 | 🞎 | 🞎 | 🞎 | 🞎 |
| I get a lot of useful information through social media sites | 🞎 | 🞎 | 🞎 | 🞎 | 🞎 |

**We are interested in how confident you feel about your future. Please indicate how true each of these statements are for you?**

|  | **Not at all true** |  | **Somewhat true** |  | **Very true** |
| --- | --- | --- | --- | --- | --- |
| I am confident I can live up to what my parents expect of me | 🞎 | 🞎 | 🞎 | 🞎 | 🞎 |
| I am confident I can live up to what my teachers expect of me | 🞎 | 🞎 | 🞎 | 🞎 | 🞎 |
| I am confident I can live up to what I expect of myself | 🞎 | 🞎 | 🞎 | 🞎 | 🞎 |
| I have a clear plan for what I hope to do next | 🞎 | 🞎 | 🞎 | 🞎 | 🞎 |

**Thank you for your time and your help!**
